# Exploring the potential of β-Cyclodextrin@Ti_3_C_2_T_x_ MXene in electrochemical chiral recognition of tyrosine enantiomers

**DOI:** 10.1007/s00604-026-07874-2

**Published:** 2026-02-23

**Authors:** Sevda Hasanova, Eda Gumus, Serdar Akbayrak, Erhan Zor

**Affiliations:** 1 Department of Nanoscience and Nanoengineering, Institute of Science, Necmettin Erbakan University, Konya, 42090 Türkiye; 2 Science and Technology Research and Application Center (BITAM), Necmettin Erbakan University, Konya, 42140 Türkiye; 3 Department of Basic Sciences, Faculty of Engineering, Necmettin Erbakan University, Konya, 42140 Türkiye; 4 Department of Science Education, A.K. Education Faculty, Necmettin Erbakan University, Konya, 42090 Türkiye

**Keywords:** Chiral recognition, Electrochemical sensor, Voltammetry, Chronoamperometry, Screen printed carbon electrode, Enantiomer, β-Cyclodextrin, MXene, Tyrosine

## Abstract

An electrochemical chiral sensor based on β-cyclodextrin@Ti_3_C_2_T_x_ MXene/screen printed carbon electrode (β-CD@MXene/SPCE) was developed for the determination of *D*- and *L*-tyrosine enantiomers for the first time. Morphological characterization of the material (β-CD@MXene) was performed using Field Emission-Scanning Electron Microscopy (FE-SEM) and Transmission Electron Microscopy (TEM) imaging, while X-ray Diffraction (XRD) and Fourier Transform Infrared Spectroscopy (FTIR) confirmed the crystalline structure and interaction between MXene and β-CD in structural characterization. To evaluate the electrochemical characterization of the modified electrodes, cyclic voltammetry (CV) and electrochemical impedance spectroscopy (EIS) techniques were applied. To investigate the electrochemical behavior of the tested *D*- and *L*-amino acid enantiomers, CV and differential pulse voltammetry (DPV) techniques were conducted. Among the tested chiral species, only *D*- and *L*-tyrosine showed distinct voltammetric signals. To investigate the electrochemical behavior of *D*- and *L*-tyrosine, CV and DPV techniques were conducted revealing an increase in oxidation peak currents with the increasing tyrosine concentration levels. The voltammetric response of racemic *D/L*-tyrosine was also examined. For real sample analysis, human serum was used in chronoamperometric measurements (10–100 µM), yielding a linear response. The LOD and LOQ values were 3.15 µM and 9.45 µM, respectively, for *D*-tyrosine, and 4.68 µM and 14.05 µM, respectively, for *L*-tyrosine in human serum samples.

**Graphical abstract:**

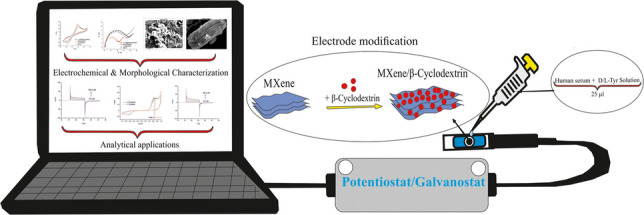

**Supplementary Information:**

The online version contains supplementary material available at 10.1007/s00604-026-07874-2.

## Introduction

Chirality, an essential physical characteristic, leads to a distinctive phenomenon where two entities are mirror reflections of one another yet cannot be perfectly aligned. A well-known example of this is the contrast between our left and right hands, which serve as separate enantiomers. Acquiring pure enantiomers is vital, as the two forms of chiral compounds can display significantly different biological and pharmacological properties. A mixture of enantiomers may result in severe complications in medical and sensor applications. Effective chiral purification techniques can substantially improve sectors such as pharmaceuticals, food production, and material development [1]. Because of the intricate composition and extremely low levels of chiral pharmaceuticals, highly precise and selective techniques are essential for environmental assessments. Early investigations into chiral analysis of environmental specimens primarily utilized gas chromatography (GC)-mass spectrometry (MS), employing either direct enantioselective approaches with a cyclodextrin-based chiral stationary phase (CSP) or indirect strategies involving derivatizing agents. Nevertheless, the application of chiral GC–MS with cyclodextrin-based CSP is frequently restricted to the examination of volatile and thermally resilient substances [2]. Chiral optical and electrochemical biosensors have garnered substantial attention in areas such as chiral detection, recognition, separation, biolabeling, and biosensing. Their potential applications span various industries, including medical treatments, cosmetic formulations, pharmaceuticals, agrochemicals, and food additives, emphasizing the importance of addressing related challenges. Electrochemical chiral sensors are extensively utilized for detecting chiral compounds due to their exceptional attributes, such as high specificity, sensitivity, cost-effectiveness, and swift analysis [3]. Every variety of chiral sensors has been developed through the strategic design of receptor molecules and host–guest interactions, enabling precise molecular recognition of enantiomers. Consequently, investigating supplementary materials is crucial to enhancing the sensor’s sensitivity, durability, and consistency [4]. Over the past few years, two-dimensional MXene materials, consisting of transition metals, carbon, and/or nitrogen, have drawn significant interest from researchers in the realm of flexible sensors. Their remarkable electrical conductivity, expansive specific surface area, exceptional hydrophilicity, and superior mechanical properties make them highly desirable in various fields including catalysis, sensors, and energy storage applications. Additionally, they can be integrated with other materials, such as carbon nanotubes, carbon fibers, and graphene, to create diverse composite structures [5]. In (bio)sensors, receptor units are immobilized onto an electrode and bound with a specific element to enable substrate transfiguration, ultimately generating biochemical signals. A transducer converts these signals into an electrical output that enables quantification of the analyte. However, when active proteins come into direct contact with the electrode surface, they lose their biological activity. Therefore, materials are selected to immobilize the active protein in a way that preserves its functionality. The use of MXenes as an immobilization matrix also facilitates direct electron transfer to the electrode, signaling a promising future for mediator-free sensors [6].

Cyclodextrins were initially identified in 1891 by Villiers and subsequently refined by Schardinger. Since their discovery, these compounds have garnered significant attention due to their high purity, water solubility, biocompatibility, and adaptability through various synthetic modifications. Additionally, their ability to encapsulate a diverse range of guest molecules via inclusion complexation makes them valuable in drug delivery, adsorption applications, and sensor development particularly in electrode modifications for highly selective electrochemical sensing [7]. β-Cyclodextrin (β-CD) has been extensively utilized in electrochemical sensing due to its ability to form host–guest complexes and its inherent chiral recognition capabilities. However, due to their water solubility, they should be stabilized on an unsolvable matrix, such as MXene, for electrochemical sensing of important bioactive materials.

Tyrosine is an aromatic amino acid found in proteins and plays a role in the synthesis of neurotransmitters (dopamine, norepinephrine, and epinephrine). It also contributes to the production of thyroid hormones and is critically important for nervous system functions. Imbalances in tyrosine levels are associated with various neurological and metabolic disorders. Tyrosine deficiency may lead to depression, dementia, and hypothyroidism, while excessive levels have been associated with Parkinson’s disease and hyperthyroidism. Understanding and regulating this balance is essential for maintaining overall physiological health [8]. Take *L*-tyrosine, for instance it has a notable impact on conditions like hyperthyroidism and may even help in managing vitiligo. In contrast, its mirror image, *D*-tyrosine, appears to influence the body’s nutritional equilibrium in a potentially suppressive way [9]. On the other hand, *D*-tyrosine is frequently employed as a molecular probe to investigate protein dynamics and structural conformations. Meanwhile, *L*-tyrosine serves as a biochemical precursor for several key neurotransmitters, including dopamine, *L*-DOPA, epinephrine, and thyroxine. Therefore, establishing a highly sensitive and efficient approach for distinguishing the enantiomers of *D*/*L*-tyrosine amino acid carries significant importance, both in theoretical studies and practical biomedical applications [10]. Therefore, the determination and discrimination of *D*- and *L*-tyrosine enantiomers are of great importance. To the best of our knowledge, the development of MXene-chiral systems remains an unexplored topic in the literature. The unique physicochemical properties of MXenes, such as high conductivity and surface functionality, have enabled their use in chiral sensing platforms, particularly for tyrosine enantiomers. Chen et al. proposed MXene/CNTs/CuMOF composites for the electrochemical detection of tyrosine [11]. The sensor exhibited a linear response in the range of 0.53–232.46 μM with a detection limit of 0.19 μM, and achieved high recovery rates in serum samples. In another study, Anh et al. synthesized highly stable nitrogen and sulfur co-doped graphene quantum dots (NSGQDs) derived from nitrogen-functionalized MXene nanosheets, and employed them as a fluorescent probe for ultra-sensitive and selective detection of 3-nitrotyrosine [12]. The probe demonstrated a wide dynamic range (0.02–150 μM) and a low detection limit of 4.2 nM in phosphate-buffered saline (PBS), along with excellent analytical performance in biological fluids, including a 7.0 nM LOD in diluted human serum. Nagrajan et al. synthesized sustainable TiO_2_ nanoparticles decorated on rGO/β-CD nanocomposites, which served as an efficient electrocatalyst for L-tyrosine detection, offering a wide dynamic range (0.01–1920 μM) and a low detection limit (7.6 nM) [13]. Karthika et al. [14] developed a CuO/β-CD nanocomposite via a sonochemical method and modified a GCE with Nafion as a binder, achieving high sensitivity (442 μA μM^−1^ cm^−2^), a low LOD (0.0082 μM), and successful detection in serum, food, and urine samples [14]. Zou et al. introduced a chiral sensing platform using 6-O-α-maltosyl-β-cyclodextrin (Mal-βCD) and BP nanosheets (BP NSs) via layer-by-layer drop casting, enabling selective recognition of tyrosine enantiomers through intermolecular hydrogen bonding, with LODs of 4.81 and 6.89 μM for *L*- and *D*-tyrosine, respectively [15]. Similarly, Zou et al. constructed a chiral composite by covalently linking amino-β-CD to single-layer graphene oxide, followed by self-assembly with BPNSs, resulting in a sensor with enhanced enantioselective recognition, particularly toward *D*-tyrosine, as evidenced by lower oxidation peak potential and higher peak current [16]. In another study, J. Zhao et al. developed a hybrid material by embedding sulfato-β-CD into macroporous carbon through simple aqueous mixing. However, there is no report combining MXenes with β-CD for investigation of the chiral recognition applications [17].

Over the past decade, our group has developed a series of graphene- and cyclodextrin-based chiral electrochemical sensors capable of discriminating various amino acid and small-molecule enantiomers, including tryptophan [18], mandelic acid [19], cystine [20], and DOPA [21] using architectures such as protein-modified graphene oxide interfaces, β-cyclodextrin–decorated reduced graphene oxide, and graphene–cyclodextrin nanohybrids. While these earlier platforms demonstrated the effectiveness of graphene derivatives and cyclodextrins as chiral selectors, the present work introduces a fundamentally different sensing strategy by integrating β-cyclodextrin with a MXene-based electrode and employing a screen-printed carbon transducer. Similar to graphene-based systems, the MXene framework offers a highly hydrophilic, conductive, and surface-rich environment that enhances host–guest interactions, facilitates faster electron transfer, and enables portable, disposable sensing formats. This new β-CD@MXene/SPCE platform therefore represents an evolution beyond our previous sensors, combining established molecular recognition capability with the distinct physicochemical advantages of MXenes to achieve improved enantioselective recognition of tyrosine enantiomers.

In this study, the proposed functionalization of Ti_3_C_2_T_x_ MXenes with CD introduces a significant innovation in the recognition of chiral molecules. By combining the natural chiral selectivity of cyclodextrins with the exceptional properties of MXenes such as high surface area, conductivity, and mechanical durability a unique platform for chiral sensors was established. This innovative approach will not only advance chiral sensor technology but also expand the potential applications of MXenes in biosensors, environmental monitoring, and biomedical fields. To achieve this discrimination, we fabricated and characterized a screen-printed carbon electrode modified with a β-CD@MXene composite and carried out a detailed electrochemical evaluation of its performance. The results demonstrate that the β-CD@MXene/SPCE functions as an effective electrochemical biosensor capable of discriminating between *D*- and *L*-tyrosine.

## Experimental

### Chemicals and instrumentation

Ti_3_AlC_2_ MAX phase was purchased from Nanografi Company in Türkiye. Hydofluoric acid (HF, 40%) and hydrochloric acid (HCl, 37%), β-cyclodextrin (β-CD, 97%) were purchased from Sigma-Aldrich. The *D*- and *L*- forms of tested amino acid derivatives (aspartic acid, cysteine, glutamine, histidine, penicillamine, phenylalanine, serine, and valine) were obtained from global chemical suppliers such as Alpha Aesar or Sigma-Aldrich, and used without any additional processing. Human serum (H4522) was purchased from Sigma. Screen-printed electrodes (SPCEs) were purchased from Metrohm Dropsens (DS 110).

Jem Jeol 2100 F Transmission electron microscopy (TEM) was used to observe MXene distribution. Field Emission SEM (ZEISS Gemini-SEM 500 and FEI Nova 430) was used to analyze the morphology of the sample. X-ray diffraction (XRD) patterns were acquired on a Rigaku Mini Flex X-ray diffractometer (radiation source Cu Kα, λ = 0.15418 nm, and scanning rate = 2 min^−1^). Fourier transform infrared spectroscopy (FTIR) analyses were performed by using Thermo Scientific/Nicolet IS20 Advanced LightDrive instrument. To achieve homogeneous dispersion of MXene-based materials in water, a Bandelin ultrasonic homogenizer was utilized. The synthesis of β-CD@MXene-based materials was performed using a Heidolph magnetic stirrer equipped with a temperature-controlled contact thermometer. For controlled vacuum drying of the materials, a CLS Scientific vacuum drying oven (CLVO-27 T) was used. During the preparation and washing processes of β-CD@MXene, a HETTICH centrifuge was employed to separate the precipitate from the solution. Electrochemical measurements were conducted using Autolab PGSTAT302N potentiostat/galvanostat, with screen-printed electrodes. Electrochemical analyses were carried out in a laboratory setting at room temperature. Prior to and following each experiment, electrode surfaces were meticulously cleaned with ultrapure water. Reproducibility was assessed using three independently prepared electrodes, while repeatability was evaluated by performing three consecutive measurements with the same electrode. Stability was examined by storing the electrode in PBS (pH 7.4) under ambient conditions and conducting measurements over a 72-h period.

### MXene synthesis

MXene was synthesized from Ti_3_AlC_2_ following a modified procedure reported in literature [22]. Briefly, 20.0 g of Ti_3_AlC_2_ was stirred with 100.0 mL HF solution (40%) for 72 h at room temperature. The resulting sample was separated by centrifugation and subsequently washed with HCl acid solution (1.0 L, 1.0 M) and distilled water (1.0 L), respectively. The obtained slurry was then dried in an oven at 90 ºC for 16 h, yielding the MXene (Ti_3_C_2_Tx) powder.

### β-CD@MXene synthesis

β-CD@MXene was synthesized according to the method presented in the literature [23]. To this aim, first, a solution containing 100.0 mg of MXene in 100.0 mL of pure water was prepared. The solution was sonicated for 30 min to ensure uniform dispersion. Then, 2.0 g of β-CD was added, followed by an additional 20 min of sonication. The mixture was heated in an oil bath at 60 °C for 4 h. Afterward, the solution was divided into two 50.0 mL tubes and centrifuged for 20 min. The supernatant was removed and replaced with 10 mL of pure water. This washing step was repeated three times, each lasting 20 min. Finally, the sample was dried in an oven at 50 °C for 24 h. The synthesis procedure of the β-CD@MXene composite was represented in Fig. S1.

### Preparation of *D/L*-tyrosine solution

*D/L*-tyrosine solution with an initial concentration of 0.01 M as stock solution was prepared in PBS. To obtain solutions with varying concentrations, specific volumes of this stock solution were diluted with PBS, resulting in final concentrations ranging approximately from 0.01 mM to 1.0 mM. The solutions of the target analytes (*D*- and *L*-tyrosine) used in the real sample analysis were prepared in 3 repetitions at concentrations of 10.0 µM, 20.0 µM, 30.0 µM, 40.0 µM, 50.0 µM, 60.0 µM, 70.0 µM, 80.0 µM and 100.0 µM, and 1% human serum was added to each solution.

### Preparation of the modified electrodes

At this stage, the prepared solutions of MXene (1.0 mg/mL), β-CD (20.0 mg/mL), and β-CD@MXene (1.0 mg/mL) were utilized. SPCEs were modified by applying 10.0 µL from each solution separately to their surfaces, followed by drying under room temperature. During the electrode modification process, a stock solution with a concentration of 1.0 mg/mL and its various dilution ratios (0.1 mg/mL, 0.2 mg/mL, and 0.4 mg/mL) were used to modify the electrode surfaces. The analyses indicated that the electrode modified at a concentration of 0.1 mg/mL exhibited superior performance compared to other dilution ratios.

## Results and discussion

### Characterization of the materials and modified electrodes

#### Structural characterization of β-CD@MXene

**Fig. 1:**
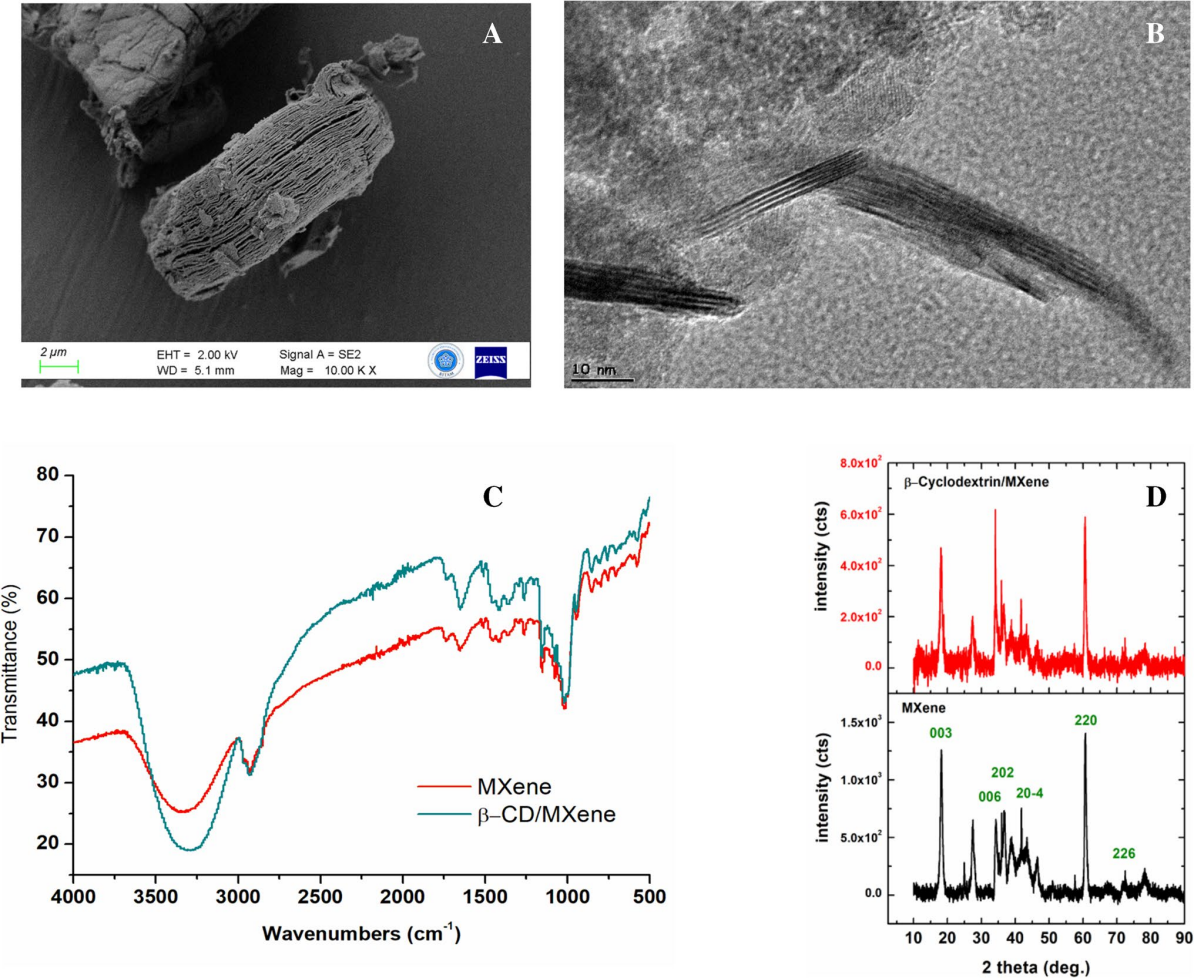
FE-SEM (**A**) and TEM images of β-CD@MXene (**B**). FTIR spectrum (**C**) and XRD patterns of MXene and β-CD@MXene samples (**D**)

β-CD is a widely employed functional intermediate in various applications owing to its unique structural property. In this study, MXene was first synthesized from the MAX phase of Ti_3_AlC_2_, and the layered morphology of the obtained MXene was clearly observed in the FE-SEM images (Fig. 1A), confirming the successful synthesis of the material. Subsequently, β-CD was anchored onto the surface of MXene via a simple functionalization procedure, enabling its application in tyrosine sensing.

The prepared β-CD@MXene composite was systematically characterized using, TEM, FE-SEM, FTIR, XRD analyses. TEM images (Fig. 1B) distinctly revealed the layered morphology of MXene along with the organic contribution of β-CD on its surface. Furthermore, FE-SEM and EDS analyses (Fig. S2-S4) corroborated that β-CD was successfully anchored onto the MXene surface, thereby validating the effectiveness of the functionalization process.

The FTIR spectrum of the MXene (Fig. 1C) showed characteristic absorption bands at 3309, 2923, 1647, and 1633 cm^−1^. The broad band at 3309 cm^−1^ is attributed to –OH stretching vibrations, which commonly arise from adsorbed water due to the subsequent aqueous washing steps. The band at 2923 cm^−1^ corresponds to C–H stretching vibrations, likely originating from surface carbon species. The peaks observed at 1647 and 1633 cm^−1^ are associated with H–O–H bending modes of physisorbed water and surface-terminated functional groups [24]. The region between 1400 and 400 cm^−1^ in the FTIR spectrum shows characteristic bond vibrations of MXene sample. Carbon–fluorine (C–F) stretching mode appears between 1400 and 1000 cm^−1^. Titanium–fluorine (Ti–F) bending vibrations can be observed in the range of 750–700 cm^−1^. Additionally, titanium–oxygen (Ti–O) bending signals locates between 650 and 550 cm^−1^; and the titanium carbide (Ti–C) vibrations are observable at lower wavenumbers [25]. The FTIR spectrum of the β-CD@MXene sample showed nearly identical features to that of the pristine MXene, with no additional distinctive peaks corresponding to β-CD. The absence of discernible β-CD signals is attributed to the strong background absorption of MXene, which can mask the characteristic C–O–C and C–H vibrations of β-CD. The XRD pattern of the as-prepared MXene displayed characteristic reflections at 2θ = 18.2º, 36.2º, 36.3º, 42.1º, 60.8º, and 72.6º, which were indexed to the (003), (006), (202), (20–4), (220), and (226) planes, respectively (Fig. 1D). These diffraction peaks match well with the reference card (PDF No. 98–061–8924) for titanium carbide, confirming that the obtained MXene sample crystallizes in a hexagonal crystal system with the R3m space group. The XRD pattern of β-CD@MXene showed no observable shifts in the peak positions in the overall diffraction pattern of MXene. The preservation of the MXene reflections indicates that β-CD incorporation does not disrupt the MXene crystal lattice. This indicates that the interaction between β-CD and MXene occurs without altering the intrinsic layered structure.

#### Electrochemical characterizations with β-CD@MXene/SPCE

**Fig. 2:**
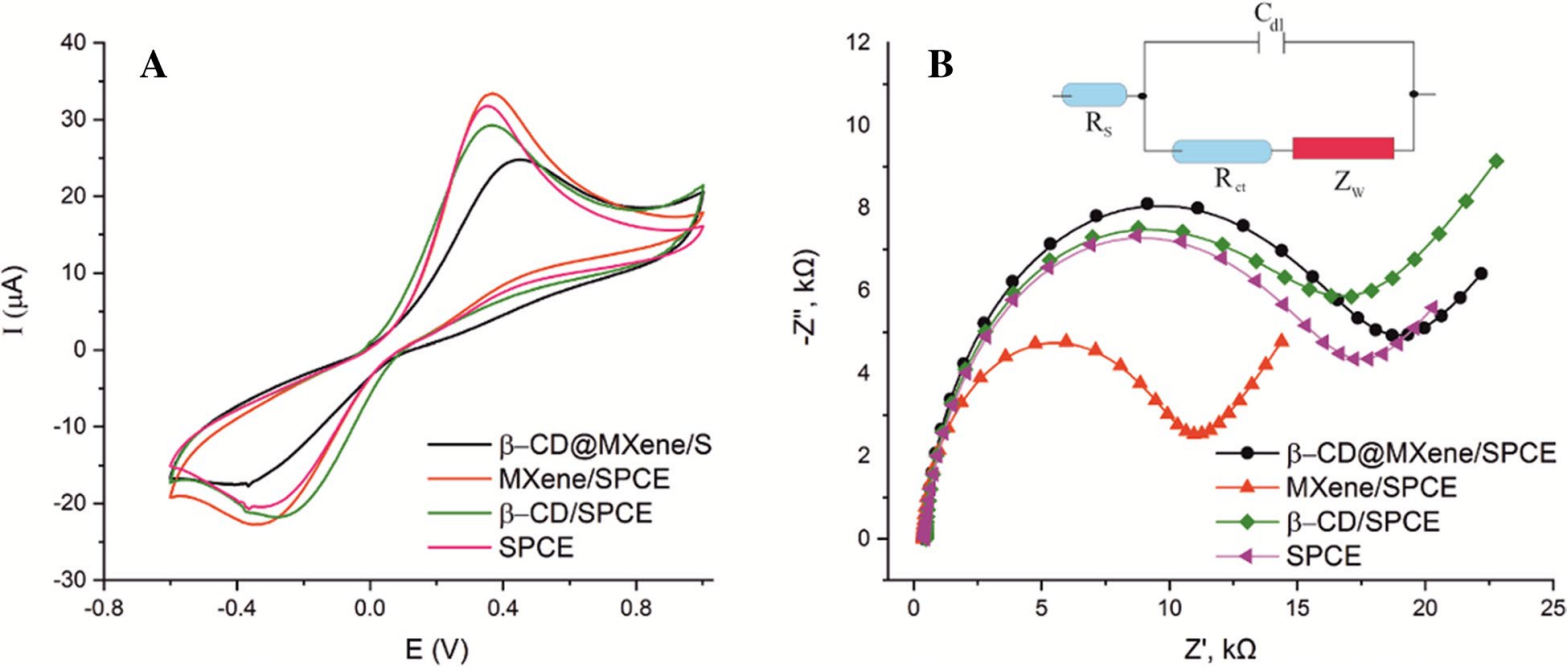
Cyclic voltammograms of SPCE, MXene/SPCE, β-CD/SPCE, and β-CD@MXene/SPCE in a solution containing 1.0 mM [Fe(CN)_6_]^3−^/^4−^ (**A**). Nyquist plots of SPCE, MXene/SPCE, β-CD/SPCE, and β-CD@MXene/SPCE in a PBS (0.1 M KCl) solution containing 1.0 mM [Fe(CN)_6_]^3−^/^4−^ (**B**). The inset shows Randles equivalent circuit model used for the analysis

To assess the electrochemical behavior of the modified electrodes obtained in this study, cyclic voltammetry (CV) and electrochemical impedance spectroscopy (EIS) were carried out in a phosphate buffer solution (PBS) containing 1.0 mM [Fe(CN)_6_] ^3−/4−^ (containing 0.1 M KCl), with the results presented in Fig. 2A. Modification of SPCE surface induces a slight change in the peak currents ratio (*I*_pa_/*I*_pc_), whereas it leads to an obvious increase in the separation of peak potentials (∆*E*_p_) value of redox couple, see Table S1. This enhancement could be ascribed to the coating of electrode surface with disrupted MXene layers and β-CD acting as an insulating layer decelerating the interfacial electron transfer.

Figure 2B shows Nyquist plots corresponding to the impedance spectra of the electrodes. The semicircular shape observed in the curves reflects the charge transfer resistance of the system. Notable variations in the semicircle radii among different electrodes were observed, highlighting the influence of surface properties on their electrochemical performance. Alike to CV results, the enhancement of the diameter on the semicircle parts in impedance spectra reveals that modification of β-CD and β-CD@MXene causes a hindering effect to electron transfer while MXene enhancing. The EIS data were fitted to the standard Randles equivalent circuit (inset of Fig. 2B), which includes the charge-transfer resistance (R_ct_) at the electrode surface. The corresponding fitted parameters are summarized in Table S1. As seen, modification of β-CD on Mxene and SPCE act as an insulating layer and increase R_ct_ values significantly.

### Electrochemical chiral recognition studies

#### Electrochemical response of β-CD@MXene/SPCE

**Fig. 3:**
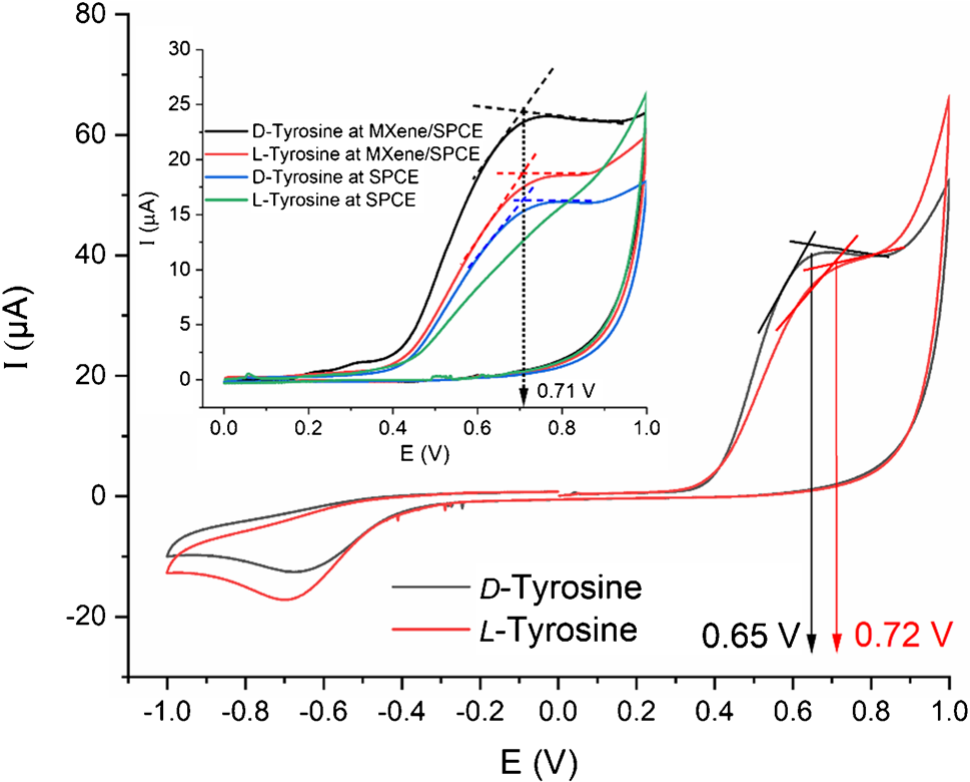
Cyclic voltammograms of the *D*- and *L*-tyrosine enantiomers at β-CD@MXene/SPCE. The inset shows the responses of bare SPCE and MXene/SPCE towards *D*- and *L*-tyrosine enantiomers. Scan rate: 100 mV/s

The enantioselective properties of β-CD@MXene were evaluated using electrochemical measurements. To examine its chiral recognition toward amino acid enantiomers, both CV and DPV techniques were employed. Among the tested amino acids, tyrosine enantiomers produced the most distinct and reproducible peak responses. As shown in Fig. 3, the β-CD@MXene/SPCE generated a clear electrochemical response upon analyte recognition, exhibiting well-defined irreversible anodic peaks at 0.65 and 0.72 V (ΔE_pa_ = 70 mV) for *D*- and *L-*tyrosine, respectively. In contrast, the bare SPCE displayed a single irreversible peak at 0.71 V, and the MXene/SPCE showed peaks at the same potential with higher current for both enantiomers, indicating no enantioselective discrimination in the anodic region of the polarization window (see inset of Fig. 3). These results can be ascribed to when β-CD is anchored to MXene, the 2D conductive surface amplifies these chiral interactions through synergistic adsorption, π–π stacking with the aromatic ring of tyrosine, and electrostatic interactions involving MXene’s surface –O, –OH, and –F terminations. The immobilized β-CD units are also conformationally restricted, which enhances stereoselective binding. This results in a different binding affinity and electron-transfer environment for each enantiomer. Thus, the enantioselectivity originates from the chiral microenvironment of β-CD, strengthened by MXene–analyte interfacial interactions, and manifested in distinct electrochemical responses. It is worth emphasizing that, unlike previous studies (Table S2), our approach directly evaluates the intrinsic peak currents and potentials of *D*- and *L*-tyrosine. This eliminates the need for any additional indicator, thereby simplifying the system and reducing uncertainties associated with interpreting the recognition mechanism. Moreover, no detectable response was observed for the other tested enantiomeric pairs (Table S3), confirming the selectivity of the β-CD@MXene/SPCE toward tyrosine enantiomers.

To gain a deeper understanding of the electroanalytical behavior of *D*- and *L*-tyrosine, voltammetric measurements were performed at various scan rates, which is a critical parameter in shaping the voltammetric response of electrochemical systems. These measurements are essential for elucidating the electron transfer mechanism and assessing whether the system is governed by diffusion or adsorption processes. The voltammetric responses of *D*- and *L*-tyrosine obtained at scan rates ranging from 10 to 150 mV/s are presented in Fig. S5. Accordingly, peak current–square root of the scan rate and log (peak current)–log (scan rate) plots were constructed based on the acquired CV curves. The linear relationship was obtained between the peak current and the square root of the scan rate over 10–150 mV/s [26]. Besides, to better understand the nature of the electrochemical process, it is not sufficient to rely solely on the Randles-Ševčík equation (peak current vs. square root of scan rate). The logarithmic dependence of peak current on scan rate allows a more direct determination of whether the process is diffusion- or adsorption-controlled. The slope of this plot provides quantitative insight into the mechanism: a slope near 1.0 indicates adsorption-controlled, and near 0.5 indicates a diffusion-controlled reaction. Within this regard, as shown in Fig. S5, the slope of the graph for *D*-tyrosine is 0.47 which shows the process is diffusion-controlled, and 0.77 for *L*-tyrosine exhibiting that both the diffusion controlled and adsorption-controlled process contribute the process [26].

**Fig. 4:**
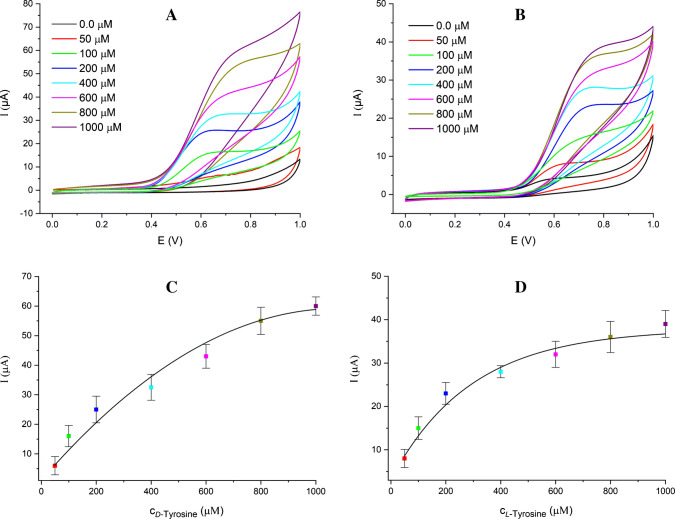
Cyclic voltammograms of *D-*tyrosine (**A**) and *L-*tyrosine (**B**) at increasing concentrations (50.0–1000.0 µM) and the calibration curve based on CV data for *D*-tyrosine (**C**) and *L-*tyrosine (**D**). Scan rate: 100 mV/s

In further experiments to examine the relationship between peak currents and the concentrations of *D*- and *L*-tyrosine, detailed voltammetric studies were conducted by increasing the concentration of the enantiomers. Figure 4A and B present the anodic cyclic voltammograms obtained in the concentration range of 50.0–1000.0 µM using samples prepared by diluting the stock solution of *D*- and *L*-tyrosine, respectively. As seen in Fig. 4C and D, the oxidation peak currents increase significantly with increasing concentration of *D*- and *L*-tyrosine, respectively.

In this study, the enantiomeric recognition of *D*- and *L*-tyrosine was achieved using a novel β-CD@MXene/SPCE-based sensor for the first time. Cyclic voltammetry measurements conducted across varying tyrosine concentrations revealed distinct peak currents for each enantiomer. Although the electrochemical profiles observed in this work resemble those reported by Chen et al. [11], and Nagarajan et al. [13], it is important to note that Chen’s study focused on general tyrosine detection using MXene-based electrodes without specifying enantiomeric differentiation, while Nagarajan’s work involved β-CD-modified platforms specifically targeting *L*-tyrosine. In contrast, the present approach enables separate and simultaneous detection of both *D*- and *L*-tyrosine enantiomers, highlighting their enhanced chiral recognition capability. However, to the best of our knowledge, this is the first report combining MXene and β-CD for chiral tyrosine sensing. The consistency of our results with previous studies confirms the reliability of the proposed method, while the integration of both materials highlights the originality and enhanced performance of the developed sensor.

**Fig. 5:**
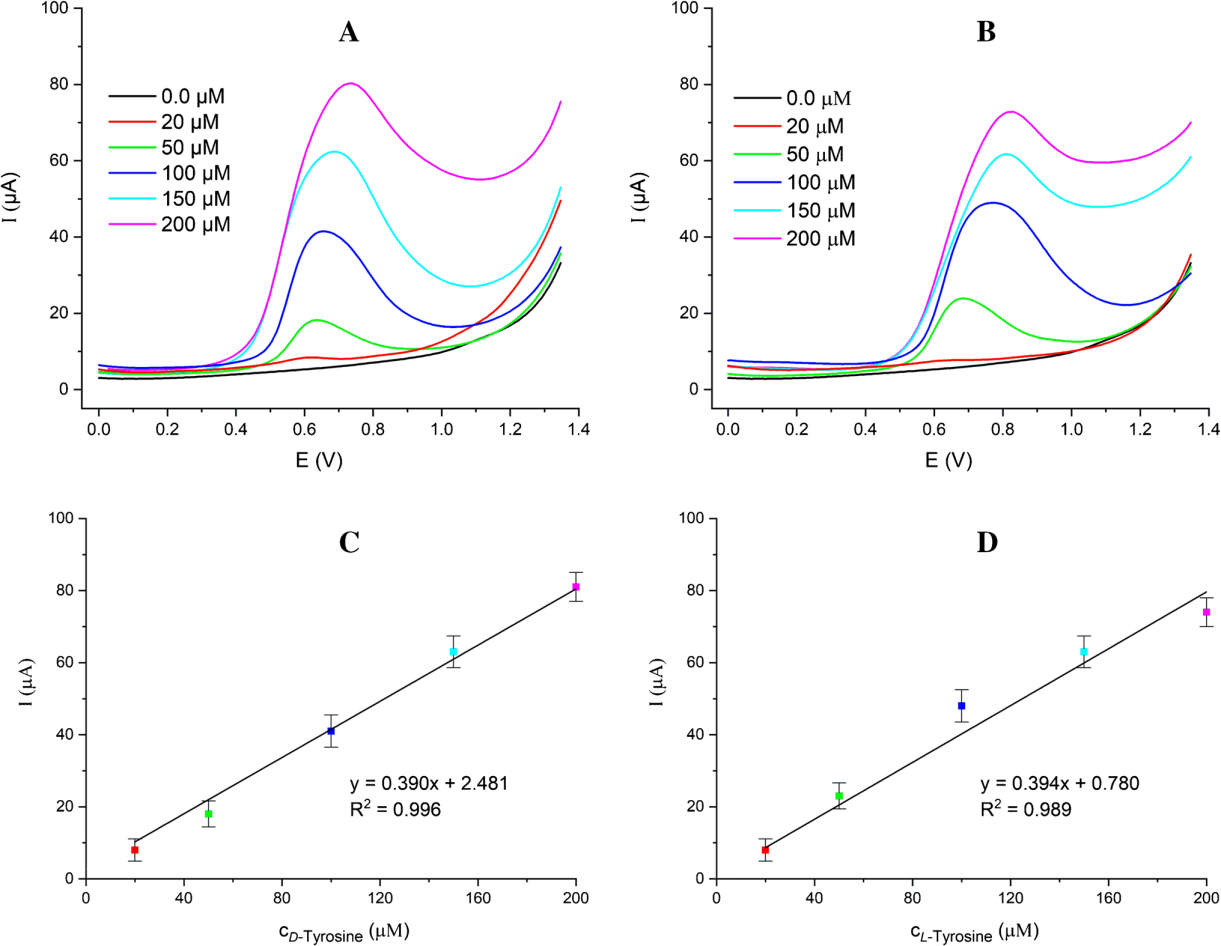
Differential pulse voltammograms of *D-*tyrosine (**A**) and *L*-tyrosine (**B**) at increasing concentrations (20.0–200.0 µM) and the calibration curves based on DPV data for *D*-tyrosine (**C**) and *L*-tyrosine (**D**). Potential step: 50 mV/s

As shown in Fig. 5A and B, DPV measurements were performed to demonstrate the electrode’s capability to detect lower concentrations with enhanced sensitivity using samples prepared by diluting *D*- and *L*-tyrosine within the concentration range of 20.0–200.0 µM. The β-CD@MXene/SPCE produced distinct and well-resolved oxidation peaks for both enantiomers across a wide concentration range. This alignment supports the accuracy of our results, while the combined use of MXene and β-CD reported in chiral tyrosine sensing underscores the novelty and analytical strength of the proposed sensor. The LOD and LOQ values were calculated as 6.47 µM and 19.62 µM for *D*-tyrosine, and were calculated as 10.63 µM and 31.92 µM for *L*-tyrosine in PBS by DPV (LOD = 3.3σ/S and LOQ = 10σ/S, where σ is the standard deviation of the blank signal and S is the slope of the regression lines in Fig. 5C and D [27]). The analytical performance parameters were summarized in Table S4.

The electrochemical behavior of the racemic mixture containing equal proportions of *D*- and *L*-tyrosine enantiomers is crucial for understanding the overall voltammetric characteristics of the system. Aiming at evaluating the performance of this sensor for racemic mixtures, simultaneous electrochemical detection of *D*- and *L*-tyrosine enantiomers was performed using a β-CD@MXene/SPCE by CV technique. A well-defined oxidation peak appeared at approximately the midpoint (0.69 V) of the *D*- and *L*-tyrosine oxidation potentials, indicating the presence of a racemic mixture in the medium (Fig. S7). This result confirms that the sensor is also applicable for analyzing racemic mixtures.

#### Real sample application: determination of *D*/*L* tyrosine in human serum samples

**Fig. 6:**
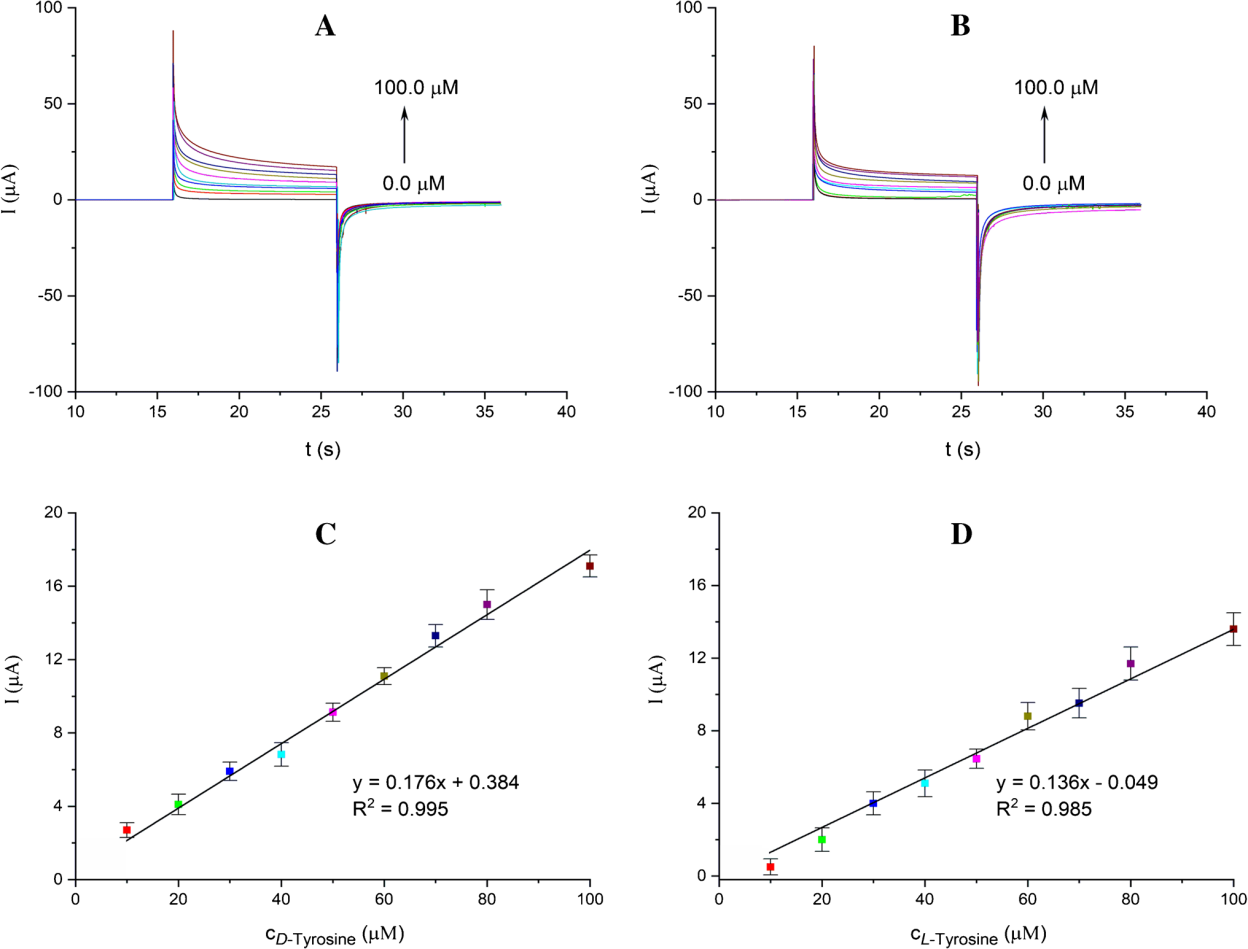
Chronoamperograms of the *D*-tyrosine (**A**) and *L*-tyrosine (**B**) at increasing concentrations (10.0–100.0 µM) in human serum using the β-CD@MXene/SPCE, and the calibration curves based on CA data for *D-*tyrosine (**C**) and *L*-tyrosine (**D**). The applied potentials are 0.00 and 0.65 V for *D*-tyrosine, 0.00 and 0.72 V for *L*-tyrosine (vs. Ag/AgCl)

As part of the real sample application, *D*/*L*-tyrosine detection was performed using the β-CD@MXene/SPCE in human serum samples. The amperometric responses of the β-CD@MXene/SPCE toward *D*- and *L*-tyrosine enantiomers (10.0–100.0 µM) were investigated using chronoamperometry (CA). CA was employed for real-sample measurements because its fixed-potential operation provides improved signal stability and robustness against matrix effects compared to potential-sweeping techniques such as CV and DPV. As shown in Fig. 6A and B, the electrode exhibited a strong amperometric response particularly for *D*-tyrosine across the increasing concentration range. The rise in oxidation currents progressed consistently with the CV and DPV results, displaying a linear trend as illustrated in Fig. 6C and D. The LOD and LOQ values were calculated as 3.15 µM and 9.45 µM for *D*-tyrosine, and 4.68 µM and 14.05 µM for *L*-tyrosine in human serum samples by CA.

To evaluate the reproducibility, voltammetric measurements were performed by successive detection of 100.0 µM *D*- and *L*-tyrosine using three different independently prepared electrodes. As shown in Fig. S6, the electrodes showed almost the same response and with the relative standard deviation (RSD) calculated to be 3.4% and 2.7% for *D*- and *L*-tyrosine, respectively. Moreover, consistency in, they offer a critical foundation for assessing the sensor’s reliability and analytical terms of repeatability. Repeatability of the β-CD@MXene/SPCE was assessed by performing three consecutive amperometric measurements, yielding RSD values of 3.1% and 3.7%, respectively. The developed β-CD-CD@MXene/SPCE sensor demonstrates robust precision, with reproducibility and repeatability that align closely with the reproducibility reported for macroporous carbon/sulfato- β-CD-cyclodextrin chiral sensors [17]. While TiO_2_@rGO/β-CD composites exhibits higher reproducibility [13], our proposed sensor exhibits a distinct operational advantage over black phosphorus-based chiral platforms, which often lack reusability due to material instability [15]. Amperometric signals of β-CD@MXene/SPCE decreased by 5.0% within 24 h, and significant amperometric response with I_max_ value of over 10 μA toward 100 µM of *D*- and *L*-tyrosine was registered even after 72 h. Although this duration is shorter than the 8-day stability (3.4% change) reported for MXene/CNTs/Cu-MOF electrodes [11], the sustained activity validates the role of MXene as a conductive scaffold. This supports the hypothesis that the large, flake-like surface of MXene prevents aggregation and stabilizes the functionalized β-CD, mirroring the protective synergy observed in carbon-embedded cyclodextrin hybrids.

## Conclusion

In this study, β-CD@MXene composite was synthesized. After the morphological, structural and electrochemical characterization of the obtained composite, it was modified onto the surface of the SPCE using a drop-casting and drying method. The resulting electrode was used for the enantiomeric discrimination of chiral compounds. In this context, the enantiomers of *D*- and *L*-tyrosine, *D*- and *L*-phenylalanine, *D*- and *L*-cysteine, *D*- and *L*-penicillamine, *D*- and *L*-histidine, *D*- and *L*-glutamine, *D*- and *L*-serine, *D*- and *L*-valine, *D*- and *L*-aspartic acid were tested. Among the chiral structures used in electrochemical studies with β-CD@MXene/SPCE, only the enantiomers of *D*- and *L*-tyrosine exhibited a distinct chiral recognition behavior. Therefore, the electrochemical chiral discrimination behavior of the β-CD@MXene/SPCE electrode toward *D*- and *L*-tyrosine enantiomers was examined in detail using CV. The DPV and CA data obtained at varying concentrations for *D*- and *L*-tyrosine showed distinct increases in oxidation peak currents. These increases were linearly correlated through calibration curves constructed for each technique. The calibration curves quantitatively revealed the relationship between analyte concentration and electrochemical response, serving as a fundamental tool for evaluating the analytical sensitivity and accuracy of the method. In the real sample application, human serum was used, and despite the low sample volume, the voltammetric responses obtained with high sensitivity confirmed the effectiveness and reliability of the electrode in biological samples. Additionally, the amperometric responses of the β-CD@MXene/SPCE electrode toward *D*- and *L*-tyrosine enantiomers in the concentration range of 10.0–100.0 µM were investigated using CA. The strong and linear signals were obtained especially for *D*-tyrosine supported the analytical consistency of the system. To conclude, β-CD@MXene represents an effective and robust receptor material that can be readily applied in the solid phase onto an electrode surface for the enantioselective recognition of *D*- and* L*-tyrosine enantiomers. In comparison with studies employing only MXene or only β-CD, the present work exhibits improved reproducibility and enhanced stability, thereby ensuring reliable long-term applicability and underscoring the originality and practical significance of this approach.

## Authors contribution

Sevda Hasanova: Methodology, Investigation, Writing—Original draft, Review \& Editing. Eda Gumus: Investigation, Writing—Original draft, Review \& Editing. Serdar Akbayrak: Methodology, Writing—Original draft, Review \& Editing. Erhan Zor: Methodology, Conceptualization, Writing—Original draft, Supervision, Review \& Editing.

## Supplementary Information

Below is the link to the electronic supplementary material.

Supplementary file1 (DOC 2523 KB)

## Data Availability

No datasets were generated or analysed during the current study.
