# Supplementary material for: Exploring the potential of β-Cyclodextrin@Ti3C2Tx MXene in electrochemical chiral recognition of tyrosine enantiomers
Source: Mikrochim Acta. 2026 Feb 23;193(3):179. doi: 10.1007/s00604-026-07874-2 (PMC12926242; doi:10.1007/s00604-026-07874-2)
Supplement: Supplementary file 1 — Supplementary file1 (DOC 2523 KB) [file 604_2026_7874_MOESM1_ESM.doc]

**Supporting Information**

**Exploring the Potential of β-Cyclodextrin@Ti_3_C_2_T_x_ MXene in Electrochemical Chiral Recognition of Tyrosine Enantiomers**

Sevda Hasanova^1^, Eda Gumus^2^, Serdar Akbayrak^2,3*^, Erhan Zor^2,4*^

*^1^Department of Nanoscience and Nanoengineering, Institute of Science, Necmettin Erbakan University, 42090, Konya, Türkiye*

*^2^Science and Technology Research and Application Center (BITAM), Necmettin Erbakan University, 42140, Konya, Türkiye*

*^3^Department of Basic Sciences, Faculty of Engineering, Necmettin Erbakan University, 42140, Konya, Türkiye*

*^4^Department of Science Education, A.K. Education Faculty, Necmettin Erbakan University, 42090 Konya, Türkiye*


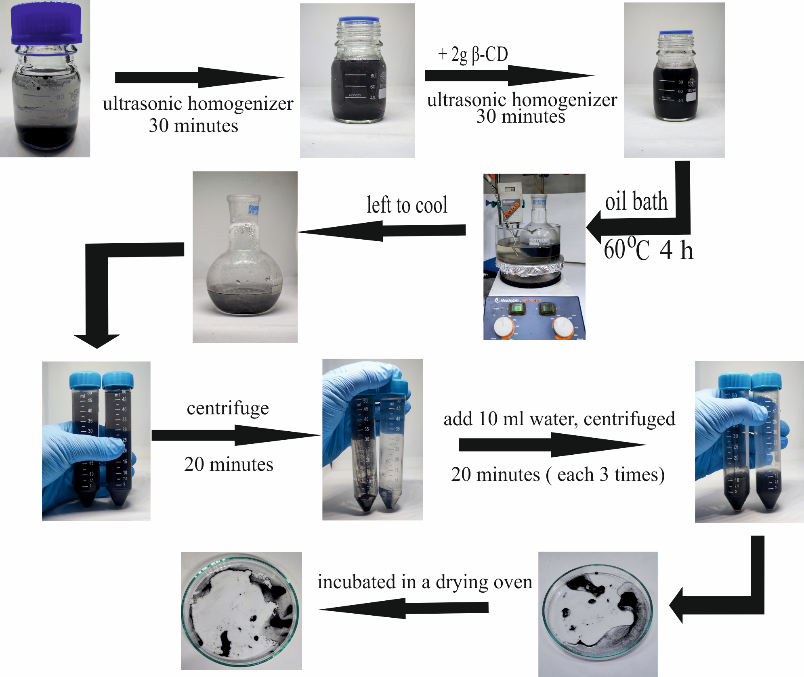


**Fig. S1.** Synthesis of the MXene/β-CD nanocomposite.

| A  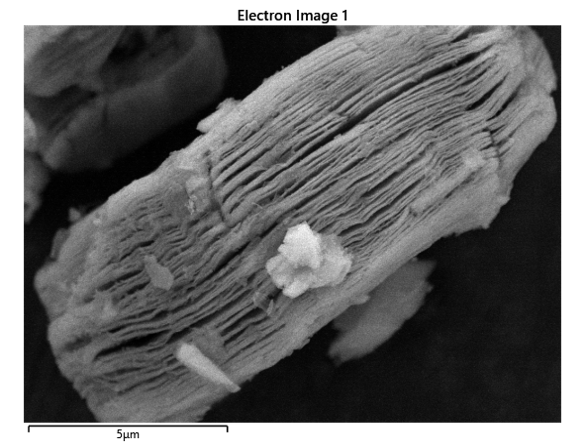 | B  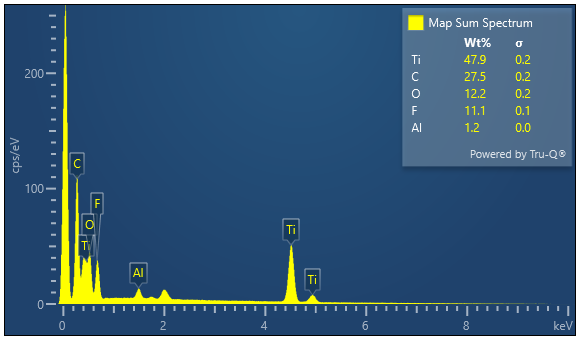 |
| --- | --- |

**Fig. S2.** FE-SEM image (A) and the corresponding EDS analysis of β-CD@MXene (B). FESEM images illustrate the surface morphology and layered structure of the MXene sample and EDS elemental mapping and corresponding sum spectrum, confirming the elemental composition of the β-CD@MXene sample.

| A  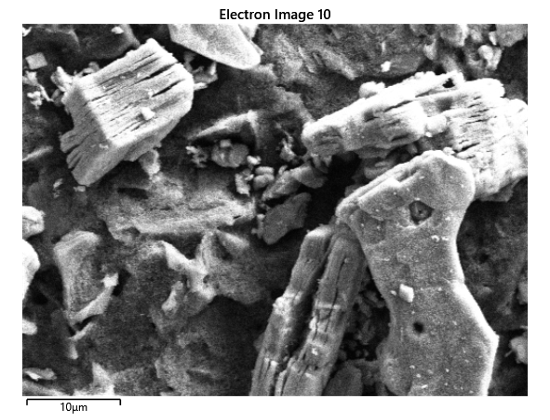 | B  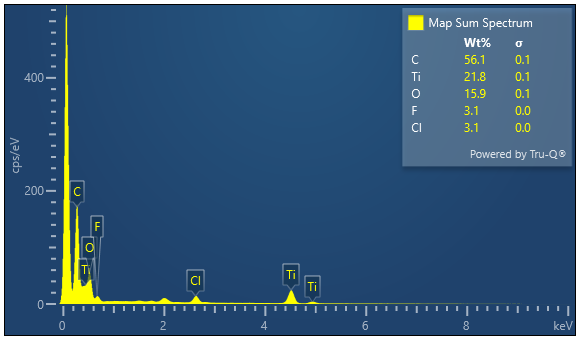 |
| --- | --- |
| C  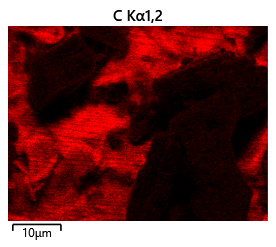 | D  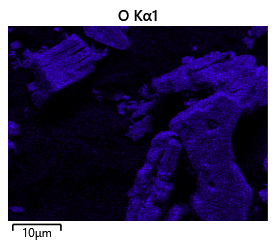 |
| E  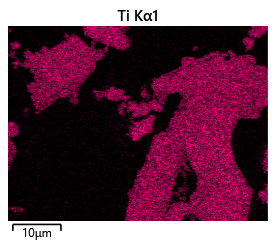 | F  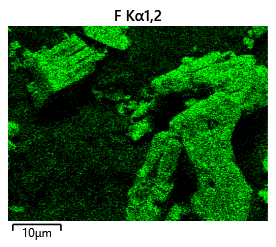 |

| G  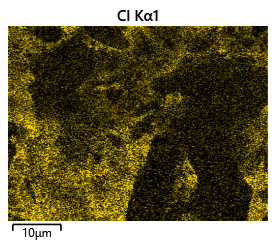 |
| --- |

**Fig. S3.** FE-SEM images and EDS analysis of the MXene/SPCE surface. (A) FE-SEM image at 10 µm scale showing the surface morphology of the MXene-modified SPCE. (B) EDS map and corresponding sum spectrum illustrating the elemental composition of the electrode surface. (C–G) Color-coded EDS elemental maps displaying the spatial distribution of carbon (C), oxygen (D), titanium (E), fluorine (F), and chlorine (G) atoms.

| A  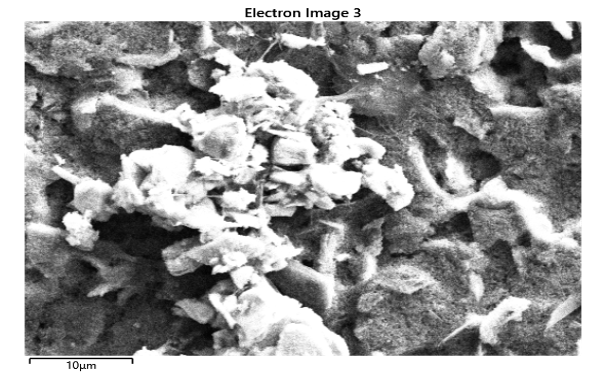 | B  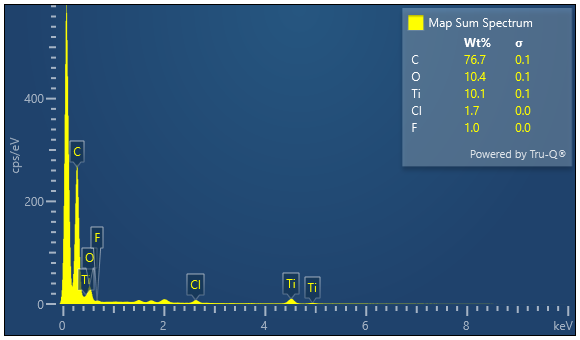 |
| --- | --- |
| C  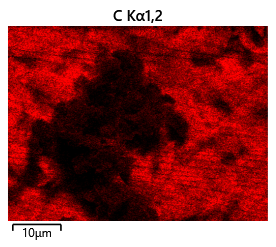 | D  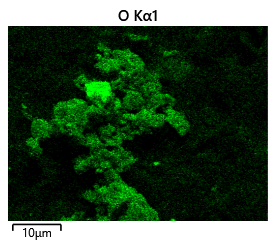 |
| E  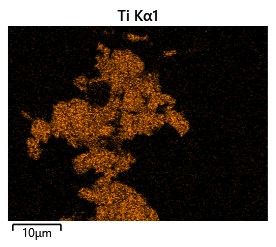 | F  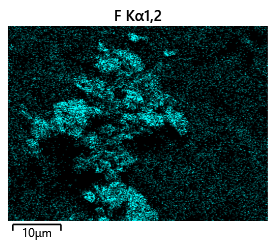 |

| G  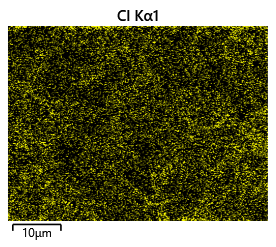 |
| --- |

**Fig. S4.** FE-SEM images and EDS analysis of the β-CD@MXene/SPCE surface. (A) FE-SEM image at 10 µm scale showing the surface morphology of the β-CD@MXene-modified SPCE. (B) EDX map and corresponding sum spectrum illustrating the elemental composition of the electrode surface. (C–G) Color-coded EDS elemental maps showing the spatial distribution of carbon (C), oxygen (D), titanium (E), fluorine (F), and chlorine (G) atoms.

| 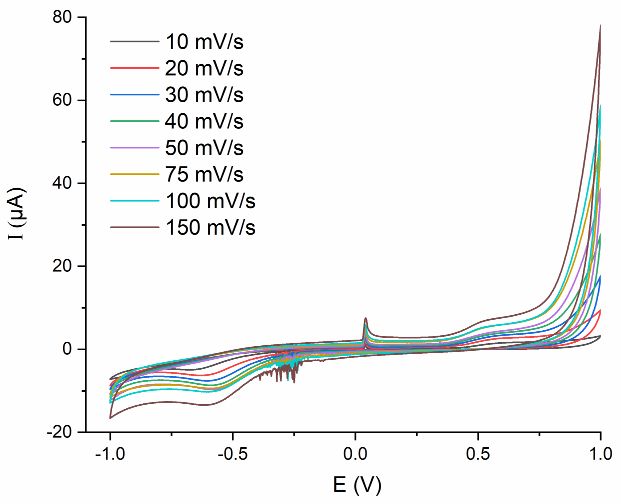 | 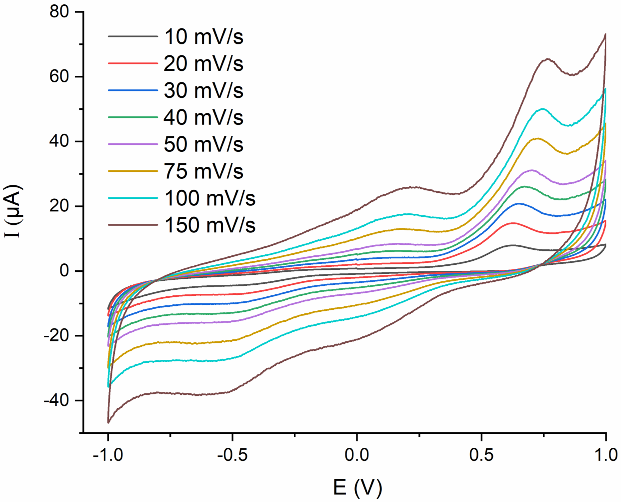 |
| --- | --- |
| 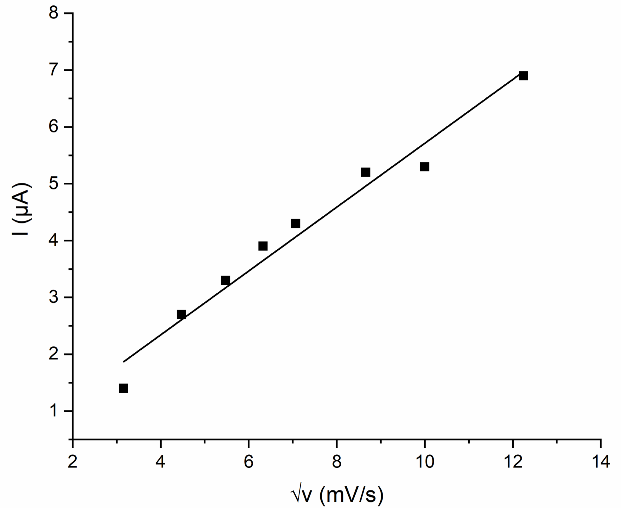 | 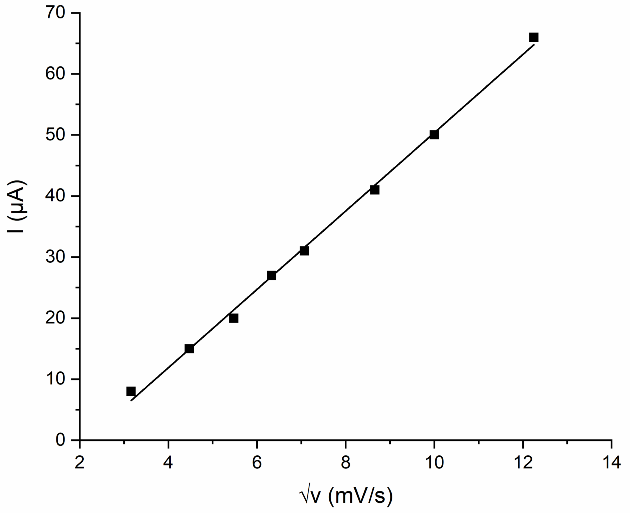 |
| 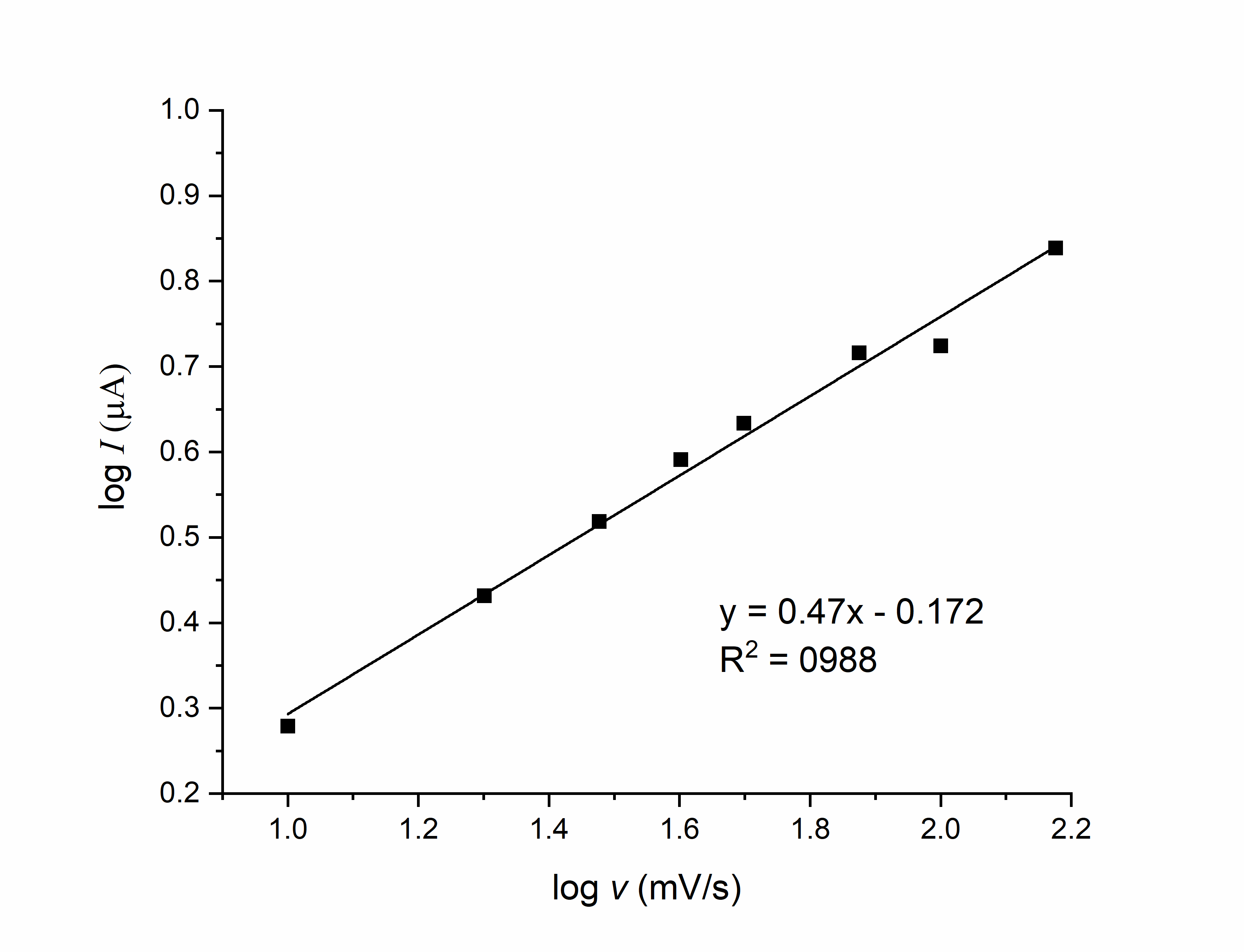 | 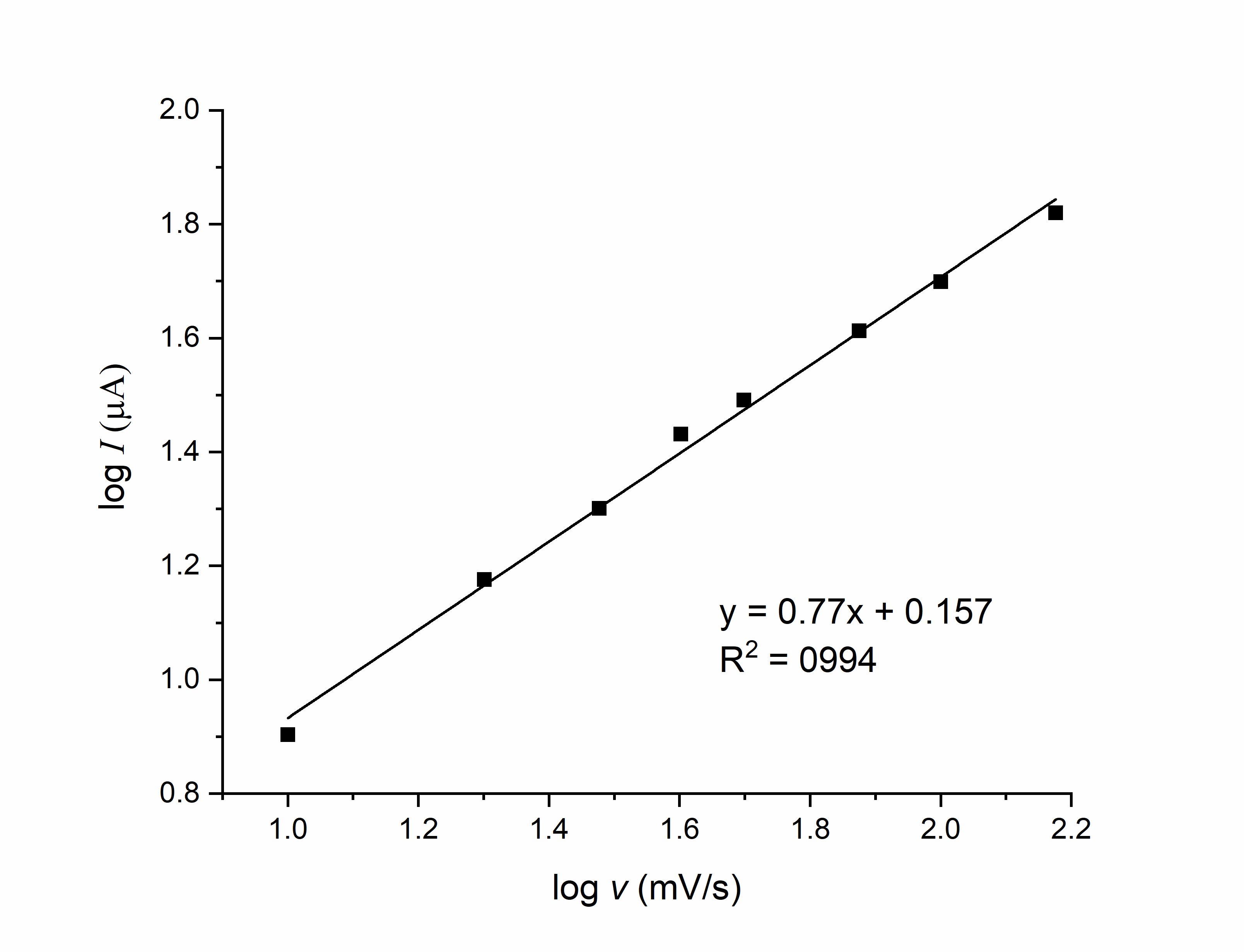 |

**Fig. S5**. Comparative presentation of CV curves obtained at different scan rates for *D*- and *L*-tyrosine using the β-CD@MXene/SPCE modified electrode, along with corresponding peak current – square root of scan rate and the log of peak current – the log of the scan rate plots.


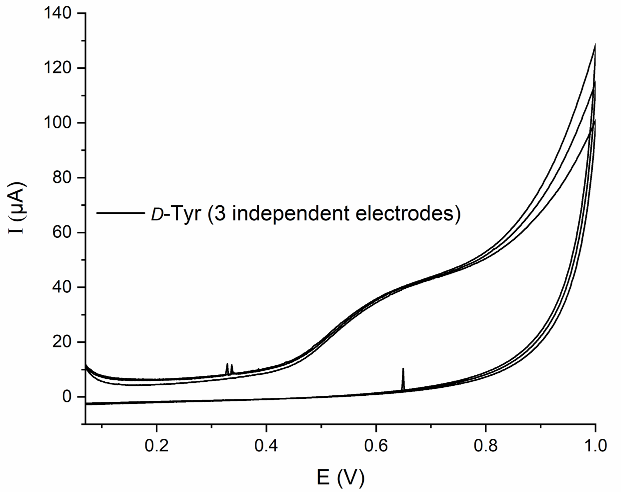

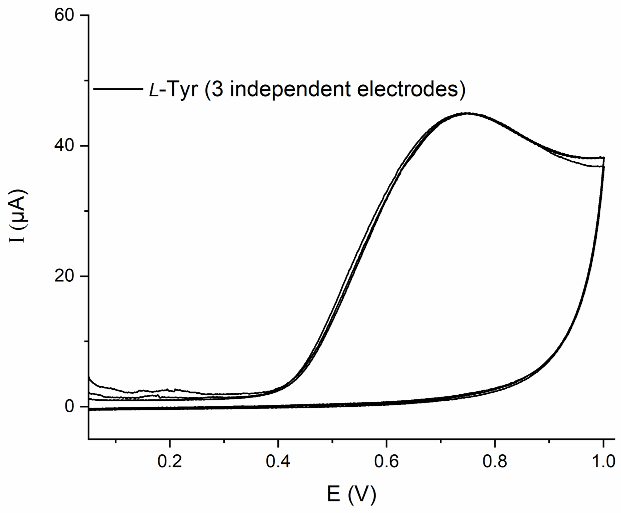


**Fig. S6.** Voltammetric curves of *D/L*-tyrosine obtained using the β-CD@MXene/SPCE. The measurements exhibit high repeatability and well-defined peak profiles. Scan rate: 100 mV/s.


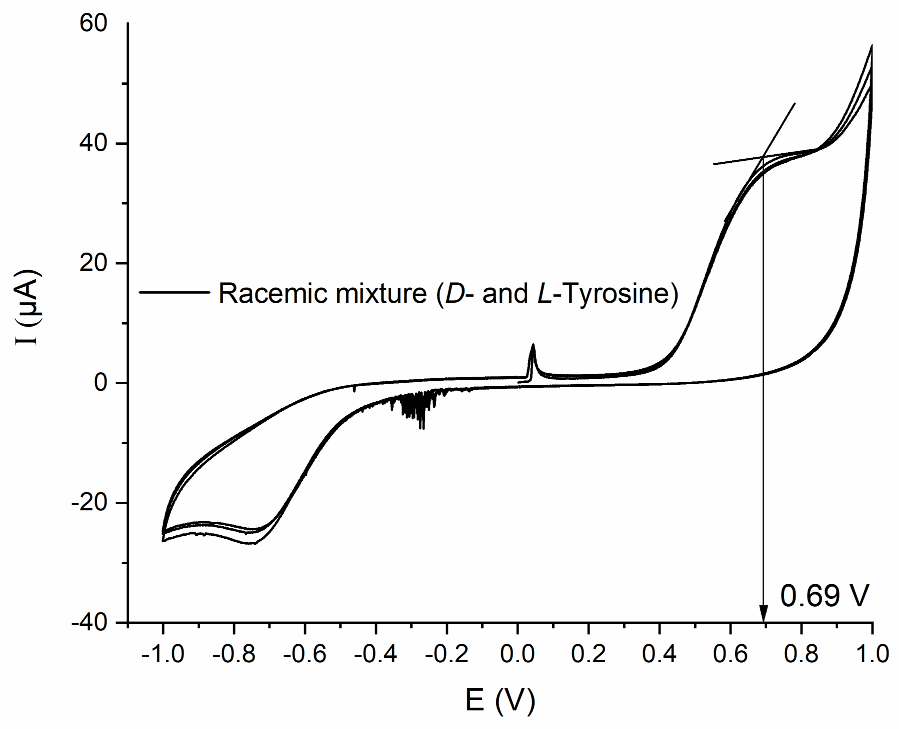


**Fig. S7.** Cyclic voltammograms of the racemic *D/L*-tyrosine mixture obtained using the β-CD@MXene/SPCE. Scan rate: 100 mV/s.

**Tables**

**Table S1.** Assessment of CV and EIS results for the tested electrodes

| Electrode | ∆*E*_p_ (V) | *I*_pa_/*I*_pc_ | *R*_ct_ (KΩ) |
| --- | --- | --- | --- |
| SPCE | 0.64 | 1.56 | 15.4 |
| MXene/SPCE | 0.68 | 1.59 | 9.58 |
| β-CD/SPCE | 0,61 | 1.36 | 14.5 |
| β-CD@MXene/SPCE | 0.79 | 1.40 | 16.6 |

**Table S2. Recent Electrochemical Chiral Biosensors for Tyrosine (Tyr) Enantiomers Using MXene and β-Cyclodextrin in the literature**

| **Target** | **Label** | **Linear Range** | **LOD** | **Enantiorecognition difference** | **Real Sample** | **Ref.** |
| --- | --- | --- | --- | --- | --- | --- |
| Tyr | MXene/CNTs/Cu-MOF/GCE | 0.53 – 232.46 μM | 0.19 μM | N/A | human serum | [1] |
| 3NTyr | NS-GQDs@MXene | 0.02 – 150 μM in PBS  0.05 – 200 μM in human serum | 4.2 nM in PBS  7 nM in human serum | N/A | human serum | [2] |
| *L*-Tyr | TiO_2_@ rGO/β-CD/GCE | 0.01 μM – 192 mM | 7.6 nM | N/A | apple juice, Tyr tablet | [3] |
| *L*-Tyr | CuO/β-CD/Nf/GCE | 0.01 – 100 μM | 0.0082 μM | N/A | blood serum, food samples, and urine samples | [4] |
| *D/L*-Tyr | Mal-βCD/BP NSs/GCE | 0.01 mM –1.0 mM | - | I*_L_*/I*_D_* = 1.51;  ΔEp = 20 mV | - | [5] |
| *D/L*-Tyr | SGO-NH_2_-βCD/BPNSs/GCE | - | - | N/A | - | [6] |
| *D/L-*Tyr | MPC-SCD/GCE | 1–500 μM | 0.20 μM (*D*-Tyr) and 0.26 μM (*L*-Tyr) | ΔI (I*_D_* − I*_L_*) = 1.12 μA | - | [7] |
| *D/L-*Tyr | β-CD@Ti_3_C_2_T_x_ MXene/SPCE | 20 – 200 µM in PBS;  10 – 100 µM in serum sample | 6.47 µM (*D*-Tyr) and 10.63 µM (*L*-Tyr) in serum sample;  3.15 µM (*D*-Tyr) and 4.68 µM (*L*-Tyr) in serum sample | ΔEp = 70 mV | commercial human serum | This study |

**N/A:** Not available; Cited articles 1, 2, 3, and 4 are electrochemical tyrosine sensors that do not address chirality at all, so they do not report enantioselectivity difference or peak potential separations for *D*- vs *L*-tyrosine.

**Table S3.** The electrochemical results for the tested chiral compounds.

| **Compound** | **Result** | **Compound** | **Result** |
| --- | --- | --- | --- |
| *L−*aspartic acid | No peak observed | *D−*aspartic acid | No peak observed |
| *L−*cysteine | No peak observed | *D−*cysteine | No peak observed |
| *L−*glutamine | No peak observed | *D−*glutamine | No peak observed |
| *L−*histidine | No peak observed | *D−*histidine | No peak observed |
| *L−*penicillamine | No peak observed | *D−*penicillamine | No peak observed |
| *L−*phenylalanine | No peak observed | *D−*phenylalanine | No peak observed |
| *L−*serine | No peak observed | *D−*serine | No peak observed |
| *L−*valine | No peak observed | *D−*valine | No peak observed |

The amino acids listed in Table S3 were chosen because they do not undergo electrooxidation within the applied potential window on the proposed sensor. This enables evaluation of nonspecific adsorption and chiral recognition at the β-cyclodextrin–functionalized interface without interference from analyte-dependent oxidation kinetics, as would occur for electroactive species such as tryptophan.

# **Table S4.** Regression data of the calibration lines for *D*- and *L*-tyrosine at β-CD@MXene/SPCE

|  | ***D*-tyrosine** | | ***L*-tyrosine** | | |
| --- | --- | --- | --- | --- | --- |
|  | **Standard**  **solution** | **Commercial**  **serum**  **sample** | **Standard solution** |  | **Commercial**  **serum**  **sample** |
| **Linearity range (µM)** | 20 - 200 | 10 -100 | 20 - 200 |  | 10 -100 |
| **Slope (µA/µM)** | 0.390 | 0.176 | 0.394 |  | 0.136 |
| **Intercept (µA)** | 2.481 | 0.384 | 0.780 |  | -0.049 |
| **SE of intercept** | 0.765 | 0.168 | 1.269 |  | 0.193 |
| **Square of correlation coefficient (*R^2^*)** | 0.996 | 0.995 | 0.989 |  | 0.985 |
| **LOD (µM)** | 6.47 | 3.15 | 10.63 |  | 4.68 |
| **LOQ (µM)** | 19.62 | 9.45 | 31.92 |  | 14.05 |
| **Repeatability of peak current (RSD%)*** | 3.1 | 3.1 | 3.7 |  | 3.7 |
| **Reproducibility of peak current (RSD%)*** | 3.4 | 3.4 | 2.7 |  | 2.7 |

*Each value is the mean of three experiments.

**References**

1. Chen J, Chen Y, Li S, et al (2022) MXene/CNTs/Cu-MOF electrochemical probe for detecting tyrosine. Carbon N Y 199:110–118. https://doi.org/10.1016/j.carbon.2022.07.021

2. Anh NTN, Huynh TV, Nguyen VT, et al (2024) MXene nanosheet-derived N, S-codoped graphene quantum dots for ultrasensitive and selective detection of 3-nitro-l-tyrosine in human serum. Anal Chim Acta 1292:342237. https://doi.org/10.1016/j.aca.2024.342237

3. Nagarajan V, Arumugam B, Annaraj J, Ramaraj SK (2022) Design of rutile nanospheres decorated rGO/β-CD nanoflakes composite: A sustainable electrocatalyst for effective non-enzymatic determination of L-Tyrosine. Sensors Actuators B Chem 351:130955. https://doi.org/10.1016/J.SNB.2021.130955

4. Karthika A, Rosaline DR, Inbanathan SSR, et al (2020) Fabrication of Cupric oxide decorated β-cyclodextrin nanocomposite solubilized Nafion as a high performance electrochemical sensor for l-tyrosine detection. J Phys Chem Solids 136:109145. https://doi.org/10.1016/J.JPCS.2019.109145

5. Zou J, Lan XW, Zhao GQ, et al (2020) Immobilization of 6-O-α-maltosyl-β-cyclodextrin on the surface of black phosphorus nanosheets for selective chiral recognition of tyrosine enantiomers. Microchim Acta 2020 18711 187:1–11. https://doi.org/10.1007/S00604-020-04606-Y

6. Zou J, Zhao G, Guan JF, et al (2021) Single-Layer Graphene Oxide-Amino-β-Cyclodextrin/Black Phosphorus Nanosheet Composites for Recognition of Tyrosine Enantiomers. ACS Appl Nano Mater 4:13329–13338. https://doi.org/10.1021/acsanm.1c02847

7. Zhao J, Cong L, Ding Z, et al (2020) Enantioselective electrochemical sensor of tyrosine isomers based on macroporous carbon embedded with sulfato-β-Cyclodextrin. Microchem J 159:105469. https://doi.org/10.1016/j.microc.2020.105469
